# Systemic and Local Regulation of Root Growth by Vascular Trehalose 6‐Phosphate is Correlated With Re‐allocation of Primary Metabolites Between Shoots and Roots

**DOI:** 10.1111/pce.70599

**Published:** 2026-05-21

**Authors:** Moritz Göbel, Jacqueline Foster, Philipp Westhoff, Noemi Skorzinski, Hannah L. Lepper, Maria F. Njo, Markus Schmid, Tom Beeckman, Miloš Tanurdžić, Anna Amtmann, Franziska Fichtner

**Affiliations:** ^1^ Heinrich Heine University Düsseldorf, Faculty of Mathematics and Natural Sciences Institute of Plant Biochemistry Düsseldorf Germany; ^2^ Cluster of Excellences on Plant Sciences (CEPLAS) Heinrich Heine University Düsseldorf Düsseldorf Germany; ^3^ School of Chemistry and Molecular Biosciences University of Queensland St. Lucia Australia; ^4^ Faculty of Mathematics and Natural Sciences, Metabolomics and Metabolism Laboratory Heinrich Heine University Düsseldorf Düsseldorf Germany; ^5^ Department of Plant Physiology Umeå Plant Science Centre, Umeå University Umeå Sweden; ^6^ Department of Plant Biotechnology and Bioinformatics Ghent University Ghent Belgium; ^7^ VIB Centre for Plant Systems Biology Ghent Belgium; ^8^ Department of Plant Biology Swedish University of Agricultural Sciences Uppsala Sweden; ^9^ School of Molecular Biosciences, College of Medical, Veterinary & Life Sciences University of Glasgow Glasgow UK

**Keywords:** resource allocation, root development, sugar partitioning, sugar signalling, trehalose 6‐phosphate

## Abstract

Trehalose 6‐phoshpate (Tre6P) is a key signalling molecule that reflects carbon status and integrates it with developmental decision making. Tre6P has been demonstrated to regulate various developmental processes, including vegetative growth, shoot branching, flowering, and root branching. Here, we investigate how vasculature‐derived Tre6P influences root system architecture by expressing heterologous Tre6P synthase or Tre6P phosphatase (TPP) specifically in the plant vasculature. Plants with elevated vascular‐derived Tre6P levels had smaller root systems, reduced sucrose levels, and lower root metabolite levels, whereas vascular TPP overexpression had the opposite effect. Using reciprocal grafting experiments, we identified shoot‐derived vascular Tre6P to be the main driver of these systemic responses. In lines with increased Tre6P in the shoot vasculature, the shoot‐to‐root metabolite ratios were consistently increased, indicating that Tre6P modulates metabolite allocation and utilisation to balance carbon partitioning between shoot and root growth. Our data further suggest that vascular Tre6P contributed to optimising the carbon‐to‐nitrogen ratio to support growth and fitness. Besides this systemic function, this study shows that root‐derived Tre6P also plays a critical local role in modulating root development. Collectively, our results demonstrate that Tre6P in the vasculature functions both systemically and locally to coordinate metabolic status with root growth and architecture.

## Summary statement

Trehalose 6‐phoshpate (Tre6P) consistently regulates root growth, with high Tre6P inhibiting and low Tre6P promoting primary and lateral root growth. It does so by altering the accumulation and allocation of many primary metabolites related to carbon and nitrogen metabolism, like sucrose and amino acids.

## 1 Introduction

To optimise their reproductive success – and thus their overall fitness – plants must continually adapt to a dynamic and ever‐changing environment. To accomplish this, they display a remarkable amount of plasticity. This applies to their shoot and root architecture. Both are essential for plant growth with the shoot performing photosynthesis and reproduction, and the roots being essential for water and nutrient uptake and anchorage. Plants coordinate organ growth with the supply of carbon and nutrients (Kudoyarova et al. 2015; Smith and Stitt 2007). To integrate both endogenous and exogenous signals and convert them into an appropriate developmental response, plants have developed a system of interconnected hormonal and sugar signalling pathways that respond to various stimuli (Roychoudhry and Kepinski 2022; Sami et al. 2019; Fichtner et al. 2024; Göbel and Fichtner 2023). Major factors modulating these dynamics are macronutrient and sugar availability of which the allocation is regulated in a systemic manner (Chen et al. 2012; Chen et al. 2016; Durand et al. 2018). Sucrose, a major product of carbon assimilation, is transported out of photosynthetic source tissues through the phloem towards actively growing sink organs. Inorganic macronutrients, however, are taken up via the roots and transported towards the shoots, where they are assimilated and used to synthesise essential metabolites like amino acids (Pratelli and Pilot 2014; Chen et al. 2016; Chiou and Lin 2011). Similar to sucrose, these metabolites are also distributed via the phloem (Lalonde et al. 2003; Santiago and Tegeder 2016). Therefore, availabilities of sucrose and other metabolites like amino acids must be coordinated in the phloem and matched with sink demand.

Acting as a major metabolic signal for sucrose availability, trehalose 6‐phoshpate (Tre6P) is a key regulator of plant metabolism and development that coordinates metabolic status with developmental decision making (Lunn et al. 2006; Yadav et al. 2014; Figueroa and Lunn 2016). The conceptual Tre6P‐sucrose nexus model explains the homoeostatic relationship between sucrose and Tre6P. According to this model Tre6P levels not only mirror sucrose levels, but changes in Tre6P levels also modulate sucrose levels thereby maintaining sucrose within an optimal concentration range for a given cell or tissue type (Yadav et al. 2014; Figueroa and Lunn 2016; Annunziata et al. 2026).

Besides being a signal for sucrose availability, Tre6P is also the intermediate of the trehalose biosynthesis pathway. Its levels are tightly controlled by trehalose‐6‐phosphate synthases (TPSs), which synthesise Tre6P from UDP‐glucose and glucose 6‐phosphate, and trehalose‐6‐phosphate phosphatases (TPPs), which dephosphorylate Tre6P to trehalose (Cabib and Leloir 1958). While there are three TPS enzymes which are expressed and catalytically active in *Arabidopsis thaliana* (TPS1, TPS2 and TPS4) (Ramon et al. 2009; Lunn 2007), TPS1 is the only catalytically active TPS expressed throughout plant development (Fichtner et al. 2020; Fichtner and Lunn 2021). As such, it is likely the sole producer of Tre6P after the seed stage. TPS1 is predominantly localised in the vasculature throughout the plant, including shoots and roots, and in meristematic shoot tissues (Fichtner et al. 2020; Wendrich et al. 2020; Fichtner 2025). As the vasculature is the main link between source and sink tissues, this places Tre6P synthesis and likely signalling at a highly strategic site for systemic signalling and allocation of sucrose and potentially other nutrients. For example, increasing Tre6P levels specifically in the vasculature of Arabidopsis results in transgenic plants with an early flowering and increased shoot branching phenotype (Fichtner et al. 2021, 2024). Many other important studies have focussed on the role of Tre6P in shoot development (Schluepmann et al. 2003; Dijken et al. 2004; Satoh‐Nagasawa et al. 2006; Wahl et al. 2013; Claeys et al. 2019; Ponnu et al. 2020), while much less is known about its function in the root (vasculature).

Alongside *TPS1*, several different *TPPs* show phloem‐specific expression (Wendrich et al. 2020; Fichtner 2025) and *TPPA*, *TPPB*, *TPPD*, *TPPG*, *TPPH*, *TPPI* and *TPPJ* have been reported to be expressed in or around lateral root (LR) primordia of developing LRs (Morales‐Herrera et al. 2023). *TPPB* has been shown to be expressed specifically within LR primordia during LR formation and to be a key factor in inhibiting LR formation by reducing Tre6P levels within LR primordia (Morales‐Herrera et al. 2023, 2024). Another study highlighted the role of *TPPI* during primary root (PR) growth showing that *tppi* mutant plants had significantly shorter primary roots (Lin et al. 2022). While the length of the meristematic zone was not affected in the *tppi* plants, the length of the elongation zone was significantly reduced (Lin et al. 2020). Transgenic lines with modified versions of the TPS1 protein also showed primary root growth defects, although the Tre6P levels in these lines did not directly correlate with the strength of the phenotype (Fichtner et al. 2020). These results suggest that besides LR development, Tre6P also functions in regulating primary root growth (Fichtner et al. 2020). However, the root‐specific roles of Tre6P derived from the vasculature, its native site of synthesis, remain to be elucidated.

In this study, we investigated the role of vasculature‐specific changes in Tre6P levels by overexpressing a bacterial *TPS* or a *TPP* from *Caenorhabditis elegans* under the control of the vasculature‐specific *GLYCINE‐DECARBOXYLASE P‐SUBUNIT A* (*GLDPA*) promoter from *Flaveria trinervia* (Fichtner et al. 2021). Root phenotyping of these lines revealed profound, opposite effects of high and low vascular Tre6P on root growth, with low Tre6P promoting and high Tre6P inhibiting root growth. Grafting experiments revealed local and systemic effects of vascular Tre6P. Significant changes in the accumulation and allocation of many primary metabolites were recorded in response to altered vascular Tre6P levels highlighting metabolic signals as potential mediators between shoot carbon status and root growth.

## 2 Materials and Methods

### 2.1 Plant Material

All lines are either in the *Arabidopsis thaliana* Columbia‐0 (Col‐0) wild‐type background and were described earlier *pGLDPA:GUS*, *pGLDPA:otsA.1*, *pGLDPA:otsA.5* and *pGLDPA:CeTPP.4* (Fichtner et al. 2021), or are in the *tps1‐1* mutant background (Eastmond et al. 2002) and were described earlier *pTPS1:otsA.2*, *pTPS1:TPS1[ΔN*_*2‐88*_ *]*, *pTPS1:GUS‐TPS1.5*, *pTPS1:TPS‐GUS.3* (Fichtner et al. 2020).

### 2.2 Generation of the CRISPR‐Cas9 Line

The CRISPR/Cas9 mutagenesis line (*CRISPR TPS1[ΔN*_*5‐90*_ *]*, see Supporting Information Figure S7 for more details), was generated as follows: A modified GreenGate system was used to assemble the vectors for CRISPR mutagenesis (Capovilla et al. 2017). Briefly, the *enhEC1.2:pEC1.1* promoter (Wang et al. 2015) was cloned into the GreenGate module A and used to drive expression of a Cas9 sequence codon‐optimised for Arabidopsis (Fauser et al. 2014) in module B, followed by the *rbcs* terminator in module C (Capovilla et al. 2017). Two single guide RNAs (sgRNAs, Supporting Information Table S1) targeting *TPS1* were cloned into modified GreenGate vectors D and E containing U6 promoters for sgRNA expression, as previously described (Capovilla et al. 2017). The final CRISPR–Cas9 construct was assembled from these modules using standard GreenGate cloning into the pGGZ003 vector (Lampropoulos et al. 2013), with mCherry expressed in the seed (*pAt2S3::mCherry:tMAS*; Gao et al. [2016]) in module F serving as a selection marker. The N‐terminal domain of the newly generated *CRISPR TPS1[ΔN*_*5‐90*_ *]* line was genotyped alongside the *pTPS1:TPS1[ΔN*_*2‐88*_ *]*, and the wild‐type *TPS1* sequence using primers specified in Supporting Information Table S2.

### 2.3 Plant Growth Conditions

Seeds were surface sterilised by washing them with 80% ethanol supplemented with 0.05% Tween‐20 for 5 min, followed by 5 min in 100% ethanol. After drying, the seeds were spotted onto ½ MS medium (Murashige and Skoog 1962) and stratified for at least 3 days at 4°C. Unless otherwise stated, the plates were placed vertically into 3D‐printed d‐Root boxes (Silva‐Navas et al. 2015) without combs and grown under LED lighting in a Polyklima M3Z‐TDL+ growth cabinet (Polyklima, Germany) with 16‐h photoperiods (with 10 min of ramping light before dawn and dusk), 100 µmol m^−2^s^−1^ light intensities, 60% humidity, and 22°C day and 18°C night temperatures. The growth under altered light regimes was tested under the same conditions with 12‐h, 16‐h and 24‐h photoperiods. For the sucrose and aspartate feeding experiment, the medium was supplemented with different combinations of 1% or 2% (w/v) of sucrose, 2 mM aspartate or 30 mM mannitol as osmotic control. For rosette phenotyping, seedlings were grown horizontally for 2 weeks before being transferred to soil and grown in 16‐h photoperiods, 150 µmol m^−2^s^−1^ light intensities, and 22°C day and 18°C night temperatures.

### 2.4 Phenotyping

For root phenotyping, square Petri dishes (12 × 12 cm) were scanned at the time indicated in each experiment using an Epson Perfection V600 Photo flatbed scanner (Epson, Japan). Root parameters were analysed using the EZ‐Rhizo2 application (https://www.psrg.org.uk/Rhizo-II.htm) (Shahzad et al. 2018). Reconstruction of averaged roots was done using the associated Root‐VIS2 programme. The same scans were used to analyse the shoot diameter, while rosette phenotyping at day 28 after sowing was performed using a Sony Alpha 7 II (Sony, Japan) camera. Both datasets were analysed using ImageJ (Version 1.54p; https://imagej.net/) to determine shoot diameters and areas. Images shown are representative of replicates within each experiment.

### 2.5 Metabolite Analysis

Shoots and roots were harvested from 12 or 14‐day old ungrafted, or 27‐day old grafted seedlings, respectively, and flash‐frozen in liquid nitrogen. Whole organ plant tissue was ground to a fine powder at liquid nitrogen temperature using a ball mill and water‐soluble metabolites were extracted as described in Lunn et al. (2006). Soluble sugars and starch were measured as described in (Stitt et al. 1989), and total amino acids were measured using the fluorescamine method (Bantan‐Polak et al. 2001). Tre6P, other phosphorylated intermediates and organic acids were measured as described in Supporting Information Method S1.

### 2.6 Gene Expression Analysis

RNA was extracted using a RNeasy Plant Mini Kit (Qiagen, Germany). Genomic DNA was removed using RQ1 RNase‐Free DNase (Promega, USA), and cDNA was synthesised using a LunaScript RT SuperMix Kit (NEB, USA). The PCR mix was prepared using a Luna Universal qPCR Master Mix (NEB), and quantitative reverse transcription polymerase chain reaction (qRT‐PCR) was performed in 96‐well microplates using a CFX Connect Real‐Time PCR Detection System (Bio‐Rad, USA). Gene expression of *TPS1* and the *TPP*s was calculated using the ΔΔCt method (*TPPC* and *TPPE* were excluded given their low expression according to the Arabidopsis eFP Browser (Winter et al. 2007) with Ct values calculated using LinRegPCR Version 2021.2 (https://medischebiologie.nl/files/) (Ruijter et al. 2009; Tuomi et al. 2010). *ACTIN* (combination of *ACT2*, *ACT7*, and *ACT8*; Supporting Information Table S3) was used for normalisation. Gene expression of *otsA* and *CeTPP* was calculated as relative expression (2^−(ΔCT)^) given the absence of these transcripts in wild‐type plants. Primers used for qRT‐PCR are given in Supporting Information Table S3.

### 2.7 Grafting Experiments

The main grafting experiments were performed following an adapted version of the Mix‐and‐match protocol (Vanderstraeten et al. 2022). Seeds were surface sterilised as described before, sown on ½ MS medium and stratified for 4 days at 4°C. The plates were then placed vertically into a growth cabinet running a 16/8 photoperiod with 70 µmol m^−2^s^−1^ irradiance and 22°C/18°C day/night temperatures for 3 days. For the fourth day they were transferred to 27°C (other settings unchanged). The grafting was performed by removing one cotyledon of each seedling and separating scions from rootstocks by cutting in the upper part of the hypocotyl. Afterwards, scions and rootstocks from different seedlings were aligned in petri dishes containing two wet Whatmann papers and two nitrocellulose membranes. Post‐grafting, the petri dishes were sealed, and the seedlings were regenerated for 6 days in a growth cabinet with a 16‐h photoperiod, a 70 µmol m^−2^s^−1^ light intensity and 22°C/18°C day/night temperatures. Successfully grafted seedlings without adventitious roots were transferred to fresh ½ MS medium and grown vertically in in 16‐h photoperiods with 100 µmol m^−2^s^−1^ light intensities and 22°C/18°C day/night temperatures. They were harvested 17 days after grafting at zeitgeber time 8 by separating the shoot and the roots following rapid quenching in liquid nitrogen for metabolite extractions, or scanned for root phenotyping 14 days after grafting. Grafting of the *pGLDPA:otsA.1* line was performed independently using a similar protocol without increasing the temperature to 27°C on the day prior to grafting. Additionally, the grafting was not performed in a sterile environment, and the seedlings were instead transferred to ½ MS medium containing 0.05% plant preservative mixture (PPM; Plant Cell Technology, USA) 7 days after grafting.

### 2.8 Microscopy

To assess the expression patterns of *TPS1* and expression driven by the *GLDPA* promoter β‐glucuronidase (GUS) staining assays were performed as described in (Fichtner et al. 2020). Harvested seedlings were placed in a 0.1 M sodium‐phosphate buffer (pH6.7), 5 mM K_3_[Fe(CN)_6_], 5 mM K_4_[Fe(CN)_6_]x3H_2_O and 1.2 mM 5‐bromo‐4‐chloro‐3‐indolyl‐b‐d‐glucuronic acid (cyclohexylammonium salt). Seedlings were vacuum infiltrated for 30 min and incubated overnight at 37°C in the dark. The tissue was destained by washing several times with 70% (vol/vol) ethanol. Stained seedlings were examined using a Nikon Eclipse Ti microscope (Nikon, Japan) fitted with a DS‐Fi2 camera and operated with the NIS‐Elements software. To analyse the expression patterns on transversal root sections, GUS assays were performed as previously described (Beeckman and Viane 2000) and GUS‐stained seedlings were subjected to fixation, dehydration, and embedding as previously described (De Smet et al. 2004). GUS‐stained sections were imaged using an Olympus BX51 microscope (Olympus, Japan).

### 2.9 Data Analysis

Data plotting and statistical analysis were performed using RStudio version 2025.05.0 + 496 (www.rstudio.com) running R version 4.4.1 (https://cran.rproject.org/). For plotting, the packages ggplot2 or ComplexHeatmap were used. Metabolite concentrations for heatmaps were scaled by row (z‐score) prior to clustering. Clustering was performed using Pearson correlation to compute the distance matrix (1 – r), followed by hierarchical clustering using Ward's minimum variance algorithm (*ward.D2*). The resulting dendrogram was used directly for visualisation in the heatmap. Statistical analysis was performed by conducting one‐way ANOVAs, followed by Fisher's LSD post‐hoc testing. To evaluate whether metabolite concentrations differ across all time points, a two‐way ANOVA was performed separately for each compound and tissue type. Statistical analyses of rescue effects of increased carbon and/or organic nitrogen supply were performed as planned contrasts on the Genotype x Condition interaction (where Condition was either medium or light regime, depending on the experiment). The resulting effect sizes represent the change in genotype difference relative to the control. Figures were compiled using Inkscape (Version 1.3.2 [091e20e, 2023‐11‐25, custom, https://inkscape.org/]).

## 3 Results

### 3.1 TPS1 is Expressed Throughout the Phloem of the Arabidopsis Root Vasculature

**Figure 1:**
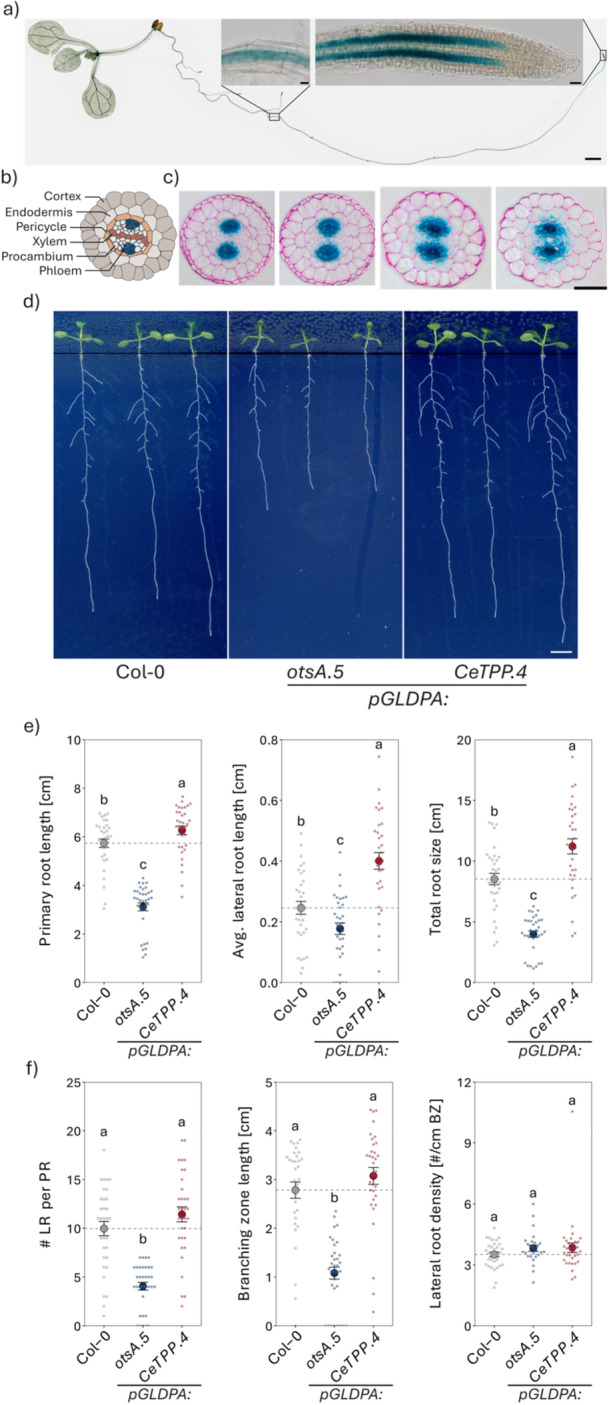
Vascular specific changes in Tre6P modify root architecture. (a) A TPS1 fusion protein tagged at the C‐terminus with the β‐GLUCURONIDASE (GUS) reporter driven by the full length *TPS1* promoter in the *tps1‐1* mutant background (*pTPS1:GUS‐TPS1.5* line) was used to analyse the expression pattern of TPS1 in Arabidopsis roots 10 days after germination (DAG) (Scale bars = 1 mm for the whole seedling; 20 µm for close‐up images of the root apical meristem and the vasculature). (b) Schematic cross section through an Arabidopsis root. (c) Transverse meristem sections of *pTPS1:GUS‐TPS1.5* seedlings 10 DAG, going from the proximal meristem to the end of the root apical meristem (from left to right). Sections were counterstained with ruthenium red (Scale bar = 50 µm). (d) Phenotypes of representative 10 DAG wild‐type (Col‐0, grey), *pGLDPA:otsA.5* (high Tre6P in the vasculature, blue), and *pGLDPA:CeTPP.4* (low Tre6P in the vasculature, red), seedlings grown vertically on ½ MS medium (Scale bar = 0.5 cm). (e) Primary root (PR), average lateral root (LR), and total root sizes (sum of the PR and all LR lengths) of plants described in (d). (f) The number of LR per PR, the length of the branching zone (BZ) and the LR density, normalised to the BZ of plants described in (d). Displayed are the mean ± SEM (large dots and error bars), with individual datapoints representing individual plants being shown as small dots (*n* ≥ 33). Letters indicate significant differences between genotypes according to a one‐way ANOVA with *post hoc* LSD testing (*p* < 0.05).

We used TPS1‐GUS and GUS‐TPS1 fusion proteins expressed under the native *TPS1* promoter and in the *tps1‐1* knockout background (Fichtner et al. 2020) to examine the precise expression pattern of the TPS1 protein within the root vasculature (Figure 1a,b,c, Supporting Information Figures S1 and S2). TPS1 was detected in the vasculature of primary and lateral roots (LR) and in the protophloem of the meristematic zone (Figure 1a). During LR development, TPS1 was detected in early stage LR primordia (LRP) up to stage III, with expression being frequently, but not always, observed in LRP of up to stage V (Supporting Information Figures S1b, S2c) (as defined by Malamy and Benfey [1997]). No expression could be detected in LRP of stages VI and VII. In stage VIII LRP and emerged LRs, TPS1 protein could be observed in the vasculature (Supporting Information Figures S1b, S2c). Using serial cross sections from the transition zone of the root meristem towards the elongation zone, we detected the TPS1 protein specifically within phloem poles, with weaker accumulation observed in parts of the procambium and pericycle (Figure 1c, Supporting Information Figure S2f).

### 3.2 Vascular Specific Alterations in Tre6P Levels Resulted in Root Growth Phenotypes

To investigate the function of Tre6P specifically within the vasculature, we used lines with alterations in Tre6P in the vasculature that were described earlier (Fichtner et al. 2021). In addition to the native Tre6P synthesis by AtTPS1 and dephosphorylation by AtTPPs, vascular tissue‐specific expression of a heterologous TPS (*otsA* from *Escherichia coli*) or TPP (*CeTPP* from *Caenorhabditis elegans*) was achieved by using the *GLYCINE‐DECARBOXYLASE P‐SUBUNIT A* (*GLDPA*) promoter from *Flaveria trinervia* (Fichtner et al. 2021). This promoter drives vascular expression very similar to the native expression pattern of *TPS1* in the vasculature, while missing the expression in developing LRP (Supporting Information Figure S3). Both overexpression lines showed profound and opposite root phenotypes (Figure 1d,e,f). Compared to Col‐0 wild‐type plants, *pGLDPA:otsA* (line #5, always referred to if not specified otherwise) plants had shorter primary roots (PRs), reduced average LR lengths, and a reduced total root length (sum of all lengths of PR and LRs) (Figure 1e). On the contrary, *pGLDPA:CeTPP* (line #4, always referred to) plants had an increased PR length, average LR length, and total root size compared to wild‐type plants (Figure 1e).

The overall decrease in PR length in *pGLDPA:otsA* plants was due to decreases in the length of both apical and branching zones (Supporting Information Figure S4). Similarly, the increase in PR length in *pGLDPA:CeTPP* plants coincided with an increase in the lengths of the apical and branching zones (Supporting Information Figure S4). *pGLDPA:otsA* plants had significantly fewer LRs per PR than wild‐type plants (Figure 1f). As they also had a smaller branching zone compared to wild‐type plants their LR density (as numbers of LR/branching zone length) was unchanged (Figure 1f). *pGLDPA:CeTPP* plants did not show significant differences compared to wild‐type plants regarding the number or density of LRs (Figure 1f). Combined, these results indicate that vascular Tre6P influences root growth along the entire root but does not directly influence LR density, which is consistent with the missing *GLDPA* promoter activity in LRPs (Supporting Information Figure S3).

Monitoring root growth over several days confirmed these observations (Supporting Information Figures S5, S6). The PR and LR lengths of the *pGLDPA:otsA* plants exhibited slower growth than the wild type, reaching similar lengths as the wild type on day 10 on day 14 for PR and on day 11 for LRs (Supporting Information Figures S5a, S6). Further, *pGLDPA:otsA* plants showed consistently fewer LRs and a reduced branching zone length, resulting in no changes in LR density (Supporting Information Figure S5b). By contrast, *pGLDPA:CeTPP* plants, showed a consistent increase in PR, LR and total root length, reaching the same length about one day earlier than wild‐type plants (Supporting Information Figure S5a). Taken together, high vascular Tre6P inhibited root growth, while lowering Tre6P in the vasculature had the opposite effect.

### 3.3 Expressing Truncated Versions of *TPS1* or *otsA* Under the Native *TPS1* Promoter Consistently Inhibits Root Growth

**Figure 2:**
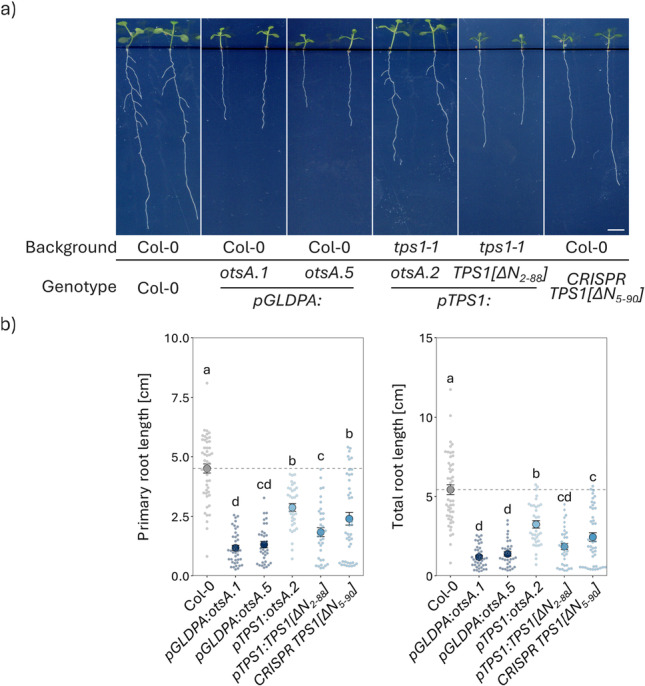
Increasing Tre6P levels by modifications in AtTPS1 also result in an inhibition of root growth. Morphological phenotypes of Arabidopsis lines expressing more active Tre6P synthase (TPS) variants. (a) Phenotypes of representative 10 days after germination (DAG) wildtype (Col‐0, grey), *pGLDPA:otsA.1* (more Tre6P in the vasculature, dark blue), *pGLDPA:otsA.5, pTPS1:otsA.2* (expression of a heterologous TPS (otsA) under the control of the *TPS1* promoter in the *tps1‐1* mutant background, light blue), *pTPS1:TPS1[ΔN*_*2‐88*_ *]* (expression of a TPS1 variant without the N‐terminal autoinhibitory domain under the control of the *TPS1* promoter in the *tps1‐1* mutant background, mid blue), and *CRISPR TPS1[ΔN*_*5‐90*_ *]* (removal of the TPS1 N‐terminal autoinhibitory domain by CRISPR/Cas9, mid blue), seedlings grown vertically on ½ MS medium (Scale bar 0.5 cm). (b) Primary (PR) and total root lengths (sum of the PR and all lateral root lengths) of the seedlings shown in a). Displayed are the mean ± SEM (large dots and error bars), with individual datapoints being shown as small dots (*n* ≥ 34). Letters indicate significant differences between genotypes according to a one‐way ANOVA with *post hoc* LSD testing (*p* < 0.05). [Color figure can be viewed at wileyonlinelibrary.com]

To exclude that the root growth phenotypes were caused mainly due to misexpression from the *GLDPA* promoter, we included additional lines with alterations in Tre6P metabolism. We used a second independent *pGLDPA:otsA* (line #*1*), a *pTPS1:otsA* (line #*2*), and a *pTPS1:TPS1[ΔN*_*2‐88*_ *]* line (Fichtner et al. 2020, 2021). The *pTPS1:otsA* and *pTPS1:TPS1[ΔN*_*2‐88*_ *]* lines were established previously and express either the *E. coli TPS* or a truncated version of *TPS1* without the N‐terminal autoinhibitory domain (missing amino acids 2 to 88, see Supporting Information Figure S7) in a *tps1‐1* mutant background. The *pTPS1:otsA* line expressing a bacterial *TPS* results in uncontrolled synthesis of Tre6P. The N‐terminal domain of TPS1 has an autoinhibitory function, deleting this domain resulted in a more active TPS1 with higher Tre6P and trehalose levels (Van Dijck et al. 2002; Fichtner et al. 2020). In addition, we generated a CRISPR‐Cas9 N‐terminal deletion line to avoid expression being dependent on the genomic context of the T‐DNA insertion (Voichek et al. 2024; Jupe et al. 2019). In this new *CRISPR TPS1[ΔN*_*5‐90*_ *]* line, the amino acids 5 to 90 of the N‐terminal domain were deleted, leading to the expression of a more active *TPS1* in its native locus (Supporting Information Figure S7). All the lines expressing either truncated versions of *TPS1* or *otsA* under the native *TPS1* promoter showed significantly decreased PR and total root lengths, supporting our observations using the two independent *pGLDPA:otsA* lines (Figure 2). The strongest reduction in PR and total root length was observed in the *pGLDPA:otsA* lines and the *pTPS1:TPS1[ΔN*_*2‐88*_ *]* line. The degree of root growth inhibition likely reflects differences in overall TPS activity: both *pGLDPA:otsA* lines overexpress the bacterial TPS in addition to the native TPS1, resulting in the highest total TPS activity and the most pronounced phenotype, whereas the other lines contain only a single active TPS with differing activity levels, leading to intermediate effects. These results confirm that the short root phenotype is not simply due to misexpression from the *GLDPA* promoter, but rather reflects altered Tre6P dynamics in the vasculature (Figures 1, 2).

### 3.4 Alterations in Vascular Tre6P Levels Modify the Correlation Between Shoot and Root Growth

**Figure 3:**
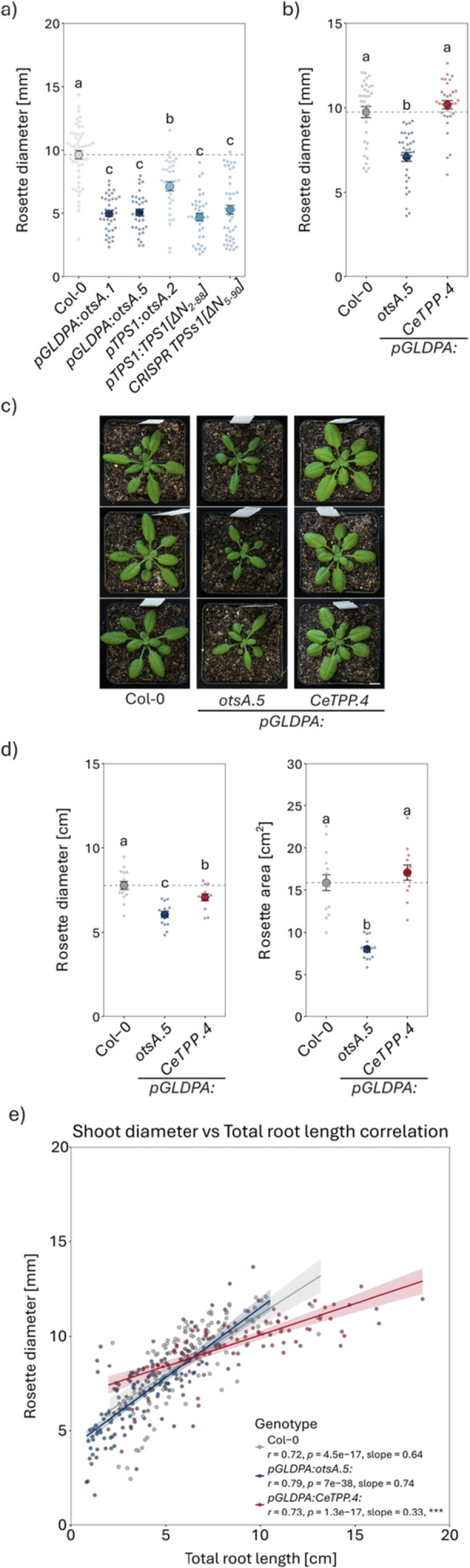
Changing vascular Tre6P levels alters rosette size and the correlation between shoot and root growth. (a) Rosette diameter of 10 days after germination (DAG) wildtype (Col‐0, grey), *pGLDPA:otsA.1* (more Tre6P in the vasculature, dark blue), *pGLDPA:otsA.5, pTPS1:otsA.2* (expression of a heterologous TPS (*otsA*) under the control of the *TPS1* promoter in the *tps1‐1* mutant background, light blue), *pTPS1:TPS1[ΔN*_*2‐88*_ *]* (expression of a TPS1 variant without the N‐terminal autoinhibitory domain under the control of the *TPS1* promoter in the *tps1‐1* mutant background, mid blue), and *CRISPR TPS1[ΔN*_*5‐90*_ *]* (removal of the TPS1 N‐terminal autoinhibitory domain by CRISPR/Cas9, mid blue), seedlings grown vertically on ½ MS medium. (b) Rosette diameter of wildtype (Col‐0, grey), *pGLDPA:otsA.5* (high Tre6P in the vasculature, blue), and *pGLDPA:CeTPP.4* (low Tre6P in the vasculature, red) seedlings grown vertically on ½ MS medium 10 DAG. (c) Representative images of wildtype (Col‐0), *pGLDPA:otsA.5*, and *pGLDPA:CeTPP.4* plants grown in long‐day conditions (16‐h photoperiod) imaged 28 days after sowing (Scale bar = 1 cm). (d) Rosette diameter and rosette area of the plants described in (c). Displayed are the mean ± SEM (large dots and error bars), with individual datapoints being shown as small dots (*n* ≥ 34 for a; *n* ≥ 33 for b; n ≥ 12 for d). (e) Pearson correlation analysis between the shoot diameter and total root size (sum of the PR and all LR lengths) in Arabidopsis wild‐type, *pGLDPA:otsA.5*, and *pGLDPA:CeTPP.4*, seedlings grown vertically on ½ MS (all data Supporting Information Figures S5a and S8 are included). Genotype‐specific Pearson correlation coefficients (r) and *p*‐values (*p*) were calculated to assess the linear relationship between the shoot diameter and total root length. Slopes were calculated using linear regression models and are displayed with 95% confidence intervals. Significant differences between the linear relationships were assessed by fitting a linear model with an interaction between the total root size and genotype. Genotype‐specific slopes were extracted using estimated marginal trends, and pairwise comparisons were performed with Col‐0 as the reference (*: *p* < 0.05; **: *p* < 0.01; ***: *p* < 0.001). [Color figure can be viewed at wileyonlinelibrary.com]

Given the tight coordination between root and shoot growth (KO and Helariutta 2017; Yang and Liu 2020; Vercruyssen et al. 2011), we also investigated how shoot growth developed over time in the different transgenic lines, using rosette diameters as a proxy for shoot size (Figure 3a,b, Supporting Information Figure S8). *pGLDPA:otsA*, *pTPS1:otsA, pTPS1:TPS1[ΔN*_*2‐88*_ *]*, and *CRISPR TPS1[ΔN*_*5‐90*_ *]* plants consistently displayed smaller rosette diameters than the wild type (Figure 3a,b, Supporting Information Figure S8). Similarly, rosette diameter and area of older plants growing on soil were significantly reduced in *pGLDPA:otsA* compared to the wild type (Figure 3c,d). In contrast, the rosette diameter of *pGLDPA:CeTPP* seedlings was similar to the rosette diameter of wild‐type seedlings (Figure 3b, Supporting Information Figure S8), while the rosette diameter of older plants grown on soil was slightly reduced compared to wild‐type plants (Figure 3c,d). However, the total rosette area was the same between *pGLDPA:CeTPP* and wild‐type plants due to differences in leaf morphology (Figure 3c,d). These shoot phenotypes are both milder but consistent with those previously observed in lines overexpressing *TPS* and *TPP* under the control of a *35S* promoter (Schluepmann et al. 2003). The stronger phenotypes observed in the *35S* lines were likely caused by pleiotropic effects of expressing *TPS* and *TPP* outside the native sites of Tre6P synthesis.

Comparing shoot with root growth by fitting a linear model to the relationship between total root size and genotype showed that in *pGLDPA:CeTPP* plants the slope of the regression line was significantly reduced compared to the wild type (0.33 compared to 0.64; Figure 3e). Given that their rosette growth was not decreased consistently, this analysis suggests that the increases in root growth in these plants are independent of their shoot growth response. In *pGLDPA:otsA* plants the correlation between shoot and root growth was not significantly different from the wild type (Figure 3e).

### 3.5 Vascular Tre6P Levels Alter Carbon Allocation

**Figure 4:**
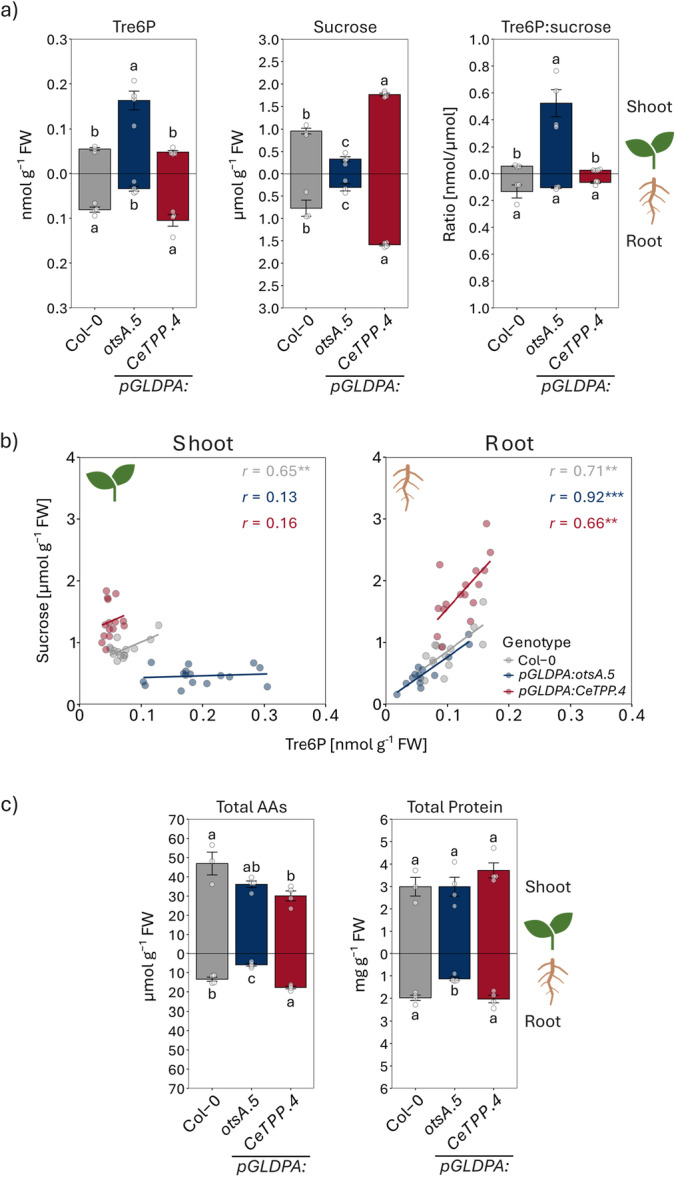
Vascular changes in Tre6P levels alter carbon allocation. Analysis of Tre6P, sucrose and other metabolites in lines expressing a heterologous TPS (otsA) or TPP (CeTPP) under the control of a vasculature‐specific promoter. (a) Tre6P and sucrose levels and Tre6P:sucrose ratios in shoots and roots of wild‐type (Col‐0, grey), *pGLDPA:otsA.5* (more vascular Tre6P, blue), *pGLDPA:CeTPP.4* (less vascular Tre6P, red) seedlings harvested 8 h after dawn (zeitgeber time [ZT] 8) from 12‐day old plants grown in a 16‐h photoperiod (100 μmol m^–2^ s^–1^ irradiance) with 22°C day/18°C night temperatures. (b) Pearson correlation analysis of sucrose and Tre6P data for shoots and roots harvested separately right after dawn (ZT0), 8 h after dawn (ZT8), immediately after the onset of darkness (ZT16), and right before dawn (ZT24). Genotype‐specific Pearson correlation coefficients (r) and p‐values were calculated to assess the linear relationship between sucrose and Tre6P levels. Significant differences between the correlations are indicated by asterisks (*: *p* < 0.05; **: *p* < 0.01; ***: *p* < 0.001). (c) Total amino acid and total protein content of the seedlings described in a). In (a) and (c), values are displayed as mean ± SEM (bar graphs and error bars), with individual datapoints being shown as dots (*n* ≥ 3). Upper panels represent shoot metabolite levels, while lower panels represent root metabolite levels. Letters indicate significant differences between genotypes according to a one‐way ANOVA with *post hoc* LSD testing (*p* < 0.05). [Color figure can be viewed at wileyonlinelibrary.com]

The altered growth responses of the *TPS* and *TPP* overexpressing lines suggested that resources might be allocated differently when Tre6P levels in the vasculature were altered. To test this hypothesis, we determined metabolic changes in their carbon metabolism. As Tre6P is a signal for sucrose availability as well as a negative feedback regulator of sucrose levels (Lunn et al. 2006; Yadav et al. 2014), it is likely that perturbing Tre6P levels would lead to changes in sucrose metabolism. We first analysed how Tre6P and sucrose levels behaved over the course of a day in shoots and roots. Figure 4 shows the Tre6P and sucrose levels, as well as the Tre6P:sucrose ratios 8 h after dawn (zeitgeber time, ZT, 8). In the *pGLDPA*:*otsA* line, shoot Tre6P levels were strongly increased alongside a significant reduction in sucrose content, leading to an increased Tre6P:sucrose ratio (Figure 4a, upper panel). The *pGLDPA:CeTPP* line, in contrast, had higher sucrose levels, while Tre6P levels remained unchanged at ZT 8 (Figure 4a, upper panel). Although not significantly different at any individual time point, Tre6P levels were significantly reduced in *pGLDPA:CeTPP* plants compared to wild‐type plants based on a two‐way ANOVA comparing Tre6P levels analysed across the diel cycle (*p* < 0.001) (Supporting Information Figure S9, Table S4). However, due to the Tre6P‐sucrose nexus (Yadav et al. 2014; Annunziata et al. 2026), a decrease in Tre6P would result in accumulation of sucrose in turn resulting in activation of Tre6P synthesis. Therefore, these plants likely sense and signal a continuous reduction in Tre6P, which is difficult to detect in our measurements at a single time point due to the activation of the native Tre6P synthesising pathway. In fact, Tre6P levels in the shoots of the *pGLDPA:CeTPP* line undergo minimal fluctuation over the course of the day, indicating that CeTPP is indeed constantly dephosphorylating Tre6P (Supporting Information Figure S9a). This was further supported by qRT‐PCR analyses, which indicated a general reduction in the expression of *TPPA*, *TPPB*, *TPPD*, *TPPF*, *TPPG*, *TPPH* and *TPPJ* in *pGLDPA:CeTPP* shoots, although none of these changes were individually significant compared to the wild type (Supporting Information Figure S10b).

Additionally, in the *pGLDPA:otsA* line, shoot Tre6P levels vary between 0.1 and 0.3 nmol per g FW while shoot sucrose levels did not fluctuate and were at around 0.4 µmol per g FW (Figure 4b). In *pGLDPA:CeTPP* shoots, sucrose levels fluctuated between 0.8 and 1.9 µmol per g FW, while Tre6P levels stayed relatively stable around 0.05 nmol per g FW. In shoots of both lines, the overexpression of *TPS* or *TPP* in the vasculature abolished the linear correlation between sucrose and Tre6P that was observed in wild‐type shoots (Figure 4b, left panel), indicating a perturbation of the Tre6P‐sucrose nexus.

Tre6P levels in the roots of the *TPS* and *TPP* vascular overexpression lines showed a very different and even opposite response to the response in the shoots. Roots of the *pGLDPA:otsA* plants contained significantly less Tre6P than wild‐type roots, while roots of *pGLDPA:CeTPP* plants contained slightly higher Tre6P levels than wild‐type plants (Figure 4a, lower panel), although both transgenes were successfully detected in root tissues albeit with lower abundances compared to shoots (Supporting Information Figure S10a). In addition, *pGLDPA:otsA* roots showed less *TPS1* transcript abundance and an increased transcript abundance of *TPPI*, likely contributing to the decrease in Tre6P (Supporting Information Figure S10b).

Sucrose levels in the roots were remarkably similar to their levels in shoots (Figure 4a, lower panel); *pGLDPA:CeTPP* plants accumulated more, while *pGLDPA:otsA* plants accumulated less sucrose compared to roots of wild‐type plants. As sucrose is supplied to the roots exclusively by photosynthetic source tissues via the phloem, root sucrose levels are expected to reflect shoot sucrose content. This was indeed observed, with lower sucrose levels in the roots of *pGLDPA:otsA* plants and higher sucrose levels in the roots of *pGLDPA:CeTPP* plants compared to wild‐type plants. According to the Tre6P‐sucrose nexus model, the altered root sucrose levels observed in the *pGLDPA* lines are likely to modulate Tre6P levels in the root. Consistently, reduced sucrose levels in the roots of *pGLDPA:otsA* plants are associated with lower Tre6P levels, whereas elevated sucrose levels in *pGLDPA:CeTPP* plants coincided with higher root Tre6P levels. This is further supported by the strong positive linear correlation between sucrose and Tre6P levels in the roots of all genotypes (Figure 4b, right panel), whereby compared to wild‐type roots, the Tre6P:sucrose ratio was unaltered in *pGLDPA:otsA* roots and higher in *pGLDPA:CeTPP* roots (Figure 4a).

An increase in Tre6P levels was consistently detected in shoots of both *pGLDPA:otsA* lines (Figure 4, S9a, S11a). In contrast, the two TPS1 N‐terminal deletion lines and the *pTPS1:otsA* line had wild‐type Tre6P levels. Nevertheless, their short‐root phenotype coincided with decreased levels of sucrose, glucose, and fructose as well as elevated Tre6P:sucrose ratios in the roots (Supporting Information Figure S11b) and, with the exception of the *CRISPR TPS1[ΔN*_*5‐90*_ *]* line, also with reduced levels of sucrose in the shoot (Supporting Information Figure S11a). These observations suggest that, although these snapshot metabolite measurements did not reveal altered Tre6P levels, Tre6P dynamics are likely affected, leading to altered sucrose responses ultimately contributing to the observed phenotypes.

### 3.6 Changes in Tre6P Levels are Accompanied by Widespread Changes in Primary Metabolism

**Figure 5:**
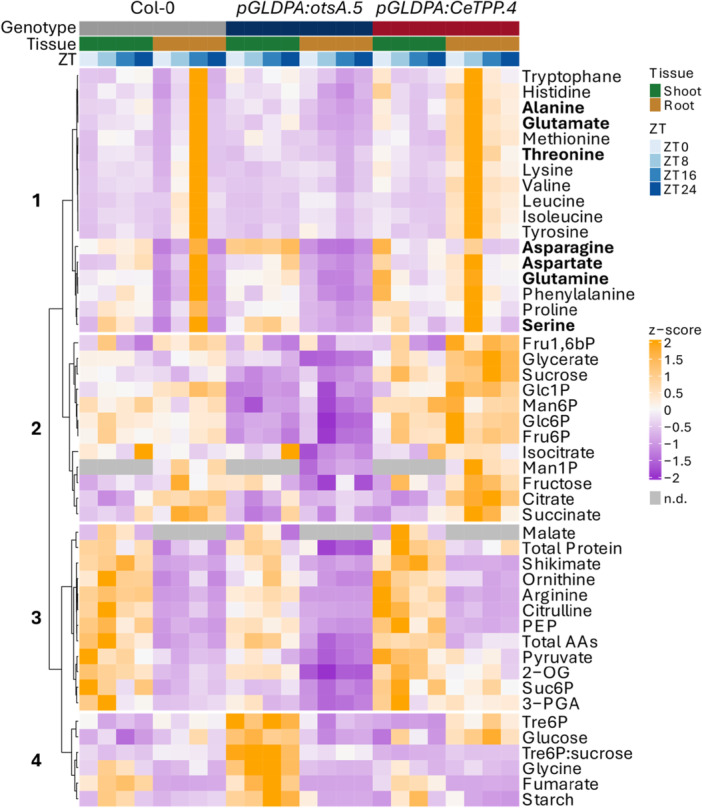
Changes in vascular Tre6P levels are accompanied by widespread changes in primary metabolism. Hierarchical clustering analysis of Tre6P and other metabolites from lines expressing a heterologous TPS (otsA) or TPP (CeTPP) under the control of a vasculature‐specific promoter (data from Supporting Information Figures S9 and S12). Shoots (green) and roots (brown) of wild‐type (Col‐0, grey), *pGLDPA:otsA.5* (more Tre6P in the vasculature, blue), *pGLDPA:CeTPP.4* (less Tre6P in the vasculature, red) seedlings were harvested separately right after dawn (zeitgeber time (ZT) 0, very light blue), 8 h after dawn (ZT8, light blue), immediately after the onset of darkness (ZT16, mid blue), and right before dawn (ZT24, dark blue) from 12‐day old plants grown in a 16‐h photoperiod (100 μmol m^–2^ s^–1^ irradiance) with 22°C day/18°C night temperatures. Z‐scores of the mean were calculated for each metabolite and are presented as a heatmap, with high and low z‐scores represented in orange and purple, respectively. Metabolites not detected in either tissue are shown in grey (n.d.). Hierarchical clustering of metabolites was performed using Pearson correlation to compute the distance matrix (1 – r), followed by Ward's minimum variance algorithm (*ward.D2*), and is represented by a dendrogram with four distinct clusters. Amino acids most abundant in the phloem sap of Arabidopsis are highlighted. [Color figure can be viewed at wileyonlinelibrary.com]

Our data indicated that alterations in vascular Tre6P levels modified nutrient allocation from shoots to roots. To obtain more detailed information we measured a large variety of primary metabolites including soluble sugars, starch, phosphorylated intermediates, tricarboxylic acid (TCA) cycle intermediates, and individual amino acids. Using hierarchical clustering we identified 4 clusters of metabolites behaving in a similar manner across genotypes, tissues, and ZTs (Figure 5).

The first cluster contains metabolites that are similar in wild‐type, *pGLDPA:otsA*, and *pGLDPA:CeTPP* shoots but that are lower in *pGLDPA:otsA*, and higher in *pGLDPA:CeTPP* roots compared to wild‐type roots. This cluster contains most of the proteogenic amino acids; especially the amino acids that are most abundant in Arabidopsis phloem sap, namely aspartate, glutamate, glutamine, serine, alanine, asparagine, and threonine (Besnard et al. 2016). In all three genotypes, shoot amino acid levels remained relatively constant over time (Supporting Information Figure S12a). In wild‐type roots, amino acids accumulated over the course of the day, reaching their highest levels at dusk (ZT16) when protein synthesis is highest (Pal et al. 2013; Ishihara et al. 2015) (Supporting Information Figure S12b). In *pGLDPA:otsA* roots this accumulation was abolished, while *pGLDPA:CeTPP* roots contained increased levels of these amino acids and showed a shift in the accumulation pattern, reaching their peak at ZT 8 (Figure 5, Supporting Information Figure S12b).

The second cluster contains metabolites that are present at similar levels in shoots and roots (on a g FW basis) and that are lower in shoots and roots of *pGLDPA:otsA* plants, and higher in shoots and roots of *pGLDPA:CeTPP* plants compared to wild‐type plants (Figure 5, Supporting Information Figure S12). This cluster contains sucrose, phosphorylated sugars, glycerate and some TCA‐cycle intermediates. Many of these compounds are primary metabolites serving as biosynthetic precursors. In both, the first and the second cluster, root metabolite levels correlate with the root phenotypes, displaying higher levels in the *pGLDPA:CeTPP* line with a larger root system, and lower levels in the *pGLDPA:otsA* line with a smaller root system.

The third cluster contains metabolites that are present at higher levels in the shoot than in the root and that are generally slightly less abundant in shoots and roots of *pGLDPA:otsA* plants, and marginally more abundant in shoots and roots of *pGLDPA:CeTPP* plants compared to wild‐type plants (Figure 4, Supporting Information Figure S12). This cluster included Suc6P (sucrose 6‐phosphate), total amino acids, total protein content, some other amino acids, pyruvate, malate, 2‐OG (2‐oxoglutarate), and 3‐PGA (3‐phosphoglycerate).

The fourth cluster contains metabolites that accumulate more in the *pGLDPA:otsA* shoots than in the shoots of *pGLDPA:CeTPP* and wild‐type plants (Figure 5, Supporting Information Figure S12). In this cluster, Tre6P groups together with glucose, glycine, fumarate, and starch. Apart from Tre6P and glycine, all these metabolites can function as a form of carbon storage (Stitt et al. 2010; Zell et al. 2010) and are reminiscent of metabolic changes induced by increased Tre6P levels (Figueroa et al. 2016). Their accumulation in *pGLDPA:otsA* shoots is likely a direct consequence of the high Tre6P levels inhibiting starch degradation (Martins et al. 2013; Ishihara et al. 2022) and pushing carbon into other storage routes (e.g. fumarate storage in the vacuole; Figueroa et al. 2016).

Taken together, these data indicate either an altered allocation or utilisation (anabolism or catabolism) of metabolites in shoots and roots induced by alterations in Tre6P levels. We therefore also explored the shoot‐to‐root ratios of the analysed metabolites (Supporting Information Figure S15). This analysis showed that while more metabolites tend to accumulate in *pGLDPA:otsA* shoots, in the *pGLDPA:CeTPP* line, metabolites preferentially accumulate in roots (Supporting Information Figure S15), correlating with the observed phenotypic responses. The *pGLDPA:otsA* plants that had smaller root systems in our analyses were previously reported to flower earlier than wild‐type plants (Fichtner et al. 2021), whereas *pGLDPA:CeTPP* plants with bigger root systems were shown to flower later than wild‐type plants (Fichtner et al. 2021). Taken together, these findings suggest that Tre6P alters root growth by changing the allocation of metabolites between shoots and roots.

### 3.7 Increased Carbon and/or Organic Nitrogen Supply Does Not Rescue Root Growth Defects in High Vascular Tre6P Lines

Our data suggests that carbon and/or organic nitrogen limitation due to low sucrose import could potentially explain the reduced root growth in lines with high vascular Tre6P (Figures 1, 2). To test this, we increased carbon supply in the *pGLDPA:otsA* line using two different approaches; increasing the photoperiod to increase the carbon availability in the shoot (Sulpice et al. 2014), and sucrose feeding through the root to increase the carbon availability directly in the root (Liu et al. 2022; Chaudhuri et al. 2008).

Increasing the photoperiod increases sucrose synthesis in the shoot (Sulpice et al. 2014), coinciding with a stimulation of root growth in both wild‐type and in *pGLDPA:otsA* plants (Supporting Information Figure S13). Both PR and total root lengths of the *pGLDPA:otsA* seedlings grown in a 20 h light/4 h dark photoperiod reached levels comparable to wild‐type seedlings grown in a 12 h light/12 h dark photoperiod. However, using a contrast on the interaction between genotype and photoperiod to estimate the difference between genotypes for each photoperiod and comparing that to the corresponding differences in neutral days (12 h light/12 h dark) showed that the total root size in the *pGLDPA:otsA* plants did not increase more strongly than that of the wild‐type in long day conditions (16 h light/8 h dark), and the differences between genotypes were even more pronounced under a 20 h light/4 h dark photoperiod (Supporting Information Table S5). These analyses indicate that, although root growth of *pGLDPA:otsA* plants can be stimulated by increased sucrose supply from the shoot, it neither fully restores a wild‐type phenotype nor reduces the differences in root size between genotypes.

We then supplied either 1% or 2% sucrose directly to the roots. Similar to the photoperiod experiment, sucrose feeding increased root size in both wild‐type and *pGLDPA:otsA* plants (Supporting Information Figure S14). While the PR and total root lengths of *pGLDPA:otsA* seedlings grown on 1% sucrose reached levels comparable to wild‐type seedlings grown without sucrose, wild‐type roots on 1% sucrose showed a similar increase, leaving the differences between genotypes unchanged. This was confirmed by analysing the interaction between genotype and medium, comparing genotype differences under each condition to the corresponding control (Supporting Information Table S5). These analyses showed that *pGLDPA:otsA* root growth was not rescued under any tested condition. In fact, the difference in total root length between *pGLDPA:otsA* and wild‐type seedlings increased under 2% sucrose relative to non‐supplemented ½ MS medium, indicating that wild‐type plants utilise high exogenous sucrose levels more effectively for root growth.

These findings indicate that Tre6P‐dependent signalling has an influence on root growth that cannot solely be attributed to alterations in sucrose allocation within the plant. Supporting the hypothesis that sucrose allocation alone is not causing the root growth phenotypes we found that *pGLDPA:otsA* plants accumulated less total amino acids in both shoots and roots and had a reduced total protein content in roots (Figure 4c). In *pGLDPA:CeTPP*, the total amino acid content of the shoots was also significantly reduced compared to wild‐type plants, but the total amino acid level of the roots was increased (Figure 4c). While roots do have the capacity to synthesise amino acids *de novo*, most amino acids are supplied to the root system by the photosynthetically active shoot, where primary nitrogen assimilation occurs and carbon skeletons are readily available from the TCA cycle (Rentsch et al. 2007; Pratelli and Pilot 2014). Like sucrose, amino acids are loaded into the phloem and transported to distant sink tissues.

To test whether a combined limitation in organic nitrogen and sucrose caused the root growth inhibition in *pGLDPA:otsA* plants, we supplied 1% sucrose together with 2 mM aspartate as a nitrogen source. This treatment consistently increased root length in both wild‐type and *pGLDPA:otsA* plants (Supporting Information Figure S14). However, as in our previous analyses, this feeding did not reduce the differences in root length between the genotypes but even increased the differences in total root length (Supporting Information Table S5). These results indicate that the root growth inhibition in *pGLDPA:otsA* lines cannot be explained solely by limitations in sucrose and organic nitrogen.

### 3.8 Shoot‐Derived Tre6P Controls Root Architecture Systemically

**Figure 6:**
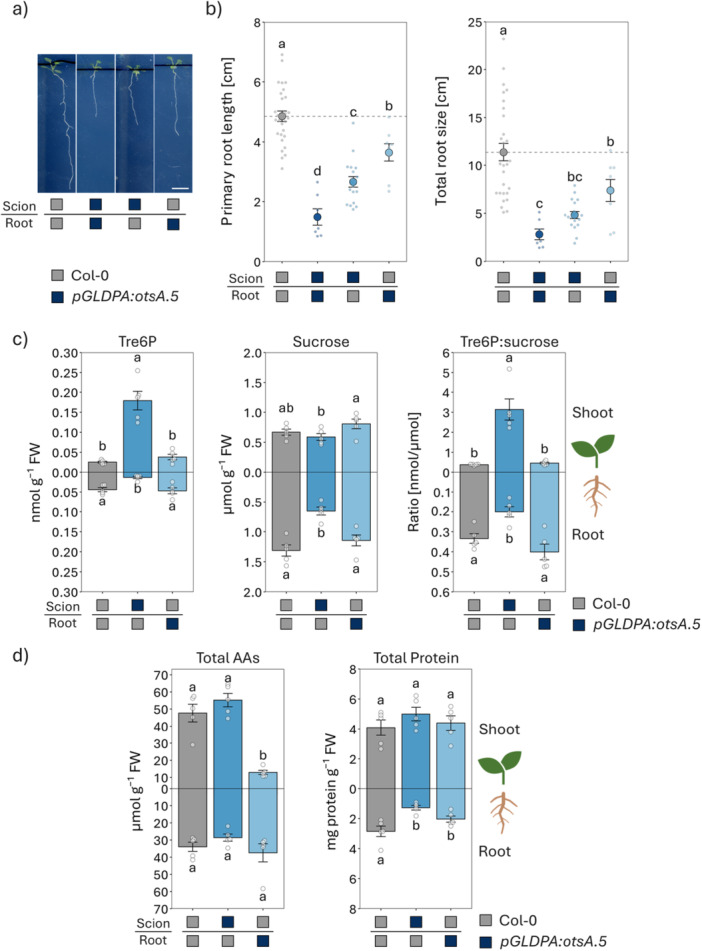
Shoot‐derived Tre6P systemically controls root architecture and metabolite allocation. Root morphology and metabolite analysis of grafted Arabidopsis seedlings with root or shoot vasculature specific expression of a bacterial TPS (otsA). (a) Phenotypes of 24‐day old representative grafted wildtype (Col‐0, grey) and *pGLDPA:otsA.5* (high Tre6P in the vasculature, shades of blue represent different graft combinations) seedlings grown vertically on ½ MS medium (Scale bar 0.5 cm). (b) Primary (PR) and total root sizes (sum of the PR and all lateral root lengths) of the seedlings shown in (a). (c) Tre6P and sucrose levels and Tre6P:sucrose ratios in shoots and roots of grafted seedlings harvested 8 h after dawn from 27‐day old plants grown in a 16‐h photoperiod (100 μmol m^–2^ s^–1^ irradiance) with 22°C day/18°C night temperatures. (d) Total amino acid (AA) and total protein levels of the same seedlings. Displayed are the mean ± SEM (large dots [b]) or bar plots ([c] and [d] and error bars), with individual datapoints being shown as small dots (b) *n* ≥ 7; (c) and (d) *n* = 5). Upper panels represent shoot metabolite levels, while lower panels represent root metabolite levels. Letters indicate significant differences between genotypes according to a one‐way ANOVA with post hoc LSD testing (*p* ≤ 0.05). [Color figure can be viewed at wileyonlinelibrary.com]

To dissect the roles of shoot and root‐derived Tre6P in modulating root growth we employed a grafting approach. We focused on the *pGLDPA:otsA* lines as we observed a stronger phenotype in these lines and had obtained independent confirmation about the Tre6P‐dependence of the phenotype in multiple lines (Figures 1, 2). As observed in ungrafted plants, *pGLDPA:otsA* (here line #*5*) homografts (*pGLDPA:otsA* scion and rootstock) exhibited the strongest inhibition of root growth, having the shortest PR and total root length among all graft combinations (Figure 6a,b). A somewhat smaller inhibition of root growth was found in heterografts with *pGLDPA:otsA* scions and wild‐type rootstocks (Figure 6a,b), while the seedlings with *pGLDPA:otsA* roots and wild‐type scions displayed the mildest inhibition of root growth (Figure 6a,b). This pattern was consistent for both PR and total root length and confirmed by a second, independent grafting experiment using the *pGLDPA:otsA* line #*1* (Supporting Information Figure S16). These data confirm that shoot‐vasculature derived Tre6P has a stronger influence on root development than root‐vasculature derived Tre6P. However, root‐vasculature derived Tre6P levels still seem to have some effect on root development as expression of *otsA* in roots alone was sufficient to significantly reduce root length (Figure 6b). As observed previously, rosette diameter in grafted *pGLDPA:otsA* seedlings correlated with root length, independent of the shoot's genotype (Supporting Information Figure S17). This provides further evidence that Tre6P is acting in a systemic manner to balance shoot and root growth.

Grafted seedlings with *pGLDPA:otsA* scions and wild‐type rootstocks showed a strong increase in Tre6P levels and the Tre6P:sucrose ratio in their shoots, while their root Tre6P levels and the Tre6P:sucrose ratio were significantly reduced compared to both wild‐type homografts and wild‐type scion *pGLDPA:otsA* rootstock heterografts (Figure 6c). The *pGLDPA:otsA* scion wild‐type rootstock heterografts further had reduced root sucrose levels (Figure 6c) and total protein contents (Figure 6d) compared to the wild‐type. In the wild‐type scion *pGLDPA:otsA* rootstock heterografts, Tre6P and sucrose levels and the Tre6P:sucrose ratio were unchanged compared to wild‐type homografts, however, a non‐significant trend for an increase in Tre6P levels was observed in the shoots (Figure 6c). These heterografts also showed a strong reduction in shoot amino acid levels and in the total protein content in the roots (Figure 6c).

When using hierarchical clustering of all measured metabolites in the grafted plants (Supporting Information Figure S18, S19), three distinct clusters were identified. The first cluster contained metabolites that were increased in the *pGLDPA:otsA* scion wild‐type rootstock heterografts compared to wild‐type homografts, while being decreased in the roots of the same plants. These were Tre6P, alongside a variety of metabolites spread across different pathways of central carbon metabolism like starch, glucose, and fumarate, which all accumulated in the high Tre6P shoots (Supporting Information Figures S18, S19). The second cluster contained most of the sugar phosphates, which in the wild‐type homografts, showed an even distribution between shoots and roots (Supporting Information Figure S18). This trend was abolished in seedlings with *pGLPDA:otsA* scions, where phosphorylated sugar levels were significantly reduced in the roots. The third cluster contained sucrose alongside all the measured TCA‐cycle intermediates. In wild‐type homografts these metabolites were more abundant in roots than in shoots, while in grafted plants with *pGLDPA:otsA* scions their levels increased in shoots and decreased in roots (Supporting Information Figure S18). The metabolic profile of wild‐type scion *pGLDPA:otsA* rootstock heterografts largely resembled that of wild‐type homografts (Supporting Information Figure S18). The notable exception was the total amino acid content, which was significantly lower in the shoots of wild‐type scion *pGLDPA:otsA* rootstock heterografts compared to the other graft combinations. Therefore, accumulation of Tre6P in the vasculature correlated with a consistent shift in metabolite accumulation in shoots and roots. This became more apparent when calculating the shoot to root ratios of these metabolites (Supporting Information Figure S20). Overall, changes in metabolism were much more pronounced in *pGLDPA:otsA* scion wild‐type rootstock heterografts and were consistent with the metabolic phenotype observed in the ungrafted *pGLDPA:otsA* seedlings, further strengthening the hypothesis that shoot‐derived Tre6P is the main driver of the shift in resource allocation and the inhibition of root growth observed.

## 4 Discussion

### 4.1 Vascular Tre6P Levels are an Important Modulator of Root Growth

Roots and shoots of plants have distinct roles in acquiring nutrients with roots extracting water and minerals from the soil and shoots fixing carbon from the atmosphere. Balancing these inputs is essential for optimal growth and development and requires steady communication between roots and shoots. Tre6P is a key signalling metabolite in flowering plants reporting on sucrose availability and acting as a negative feedback regulator of sucrose levels (Yadav et al. 2014; Fichtner and Lunn 2021). This means that in response to changes in sucrose levels, Tre6P levels also change, resulting in metabolic and developmental changes, which in turn alter sucrose levels. This homoeostatic relationship has been described as the Tre6P‐sucrose nexus (Yadav et al. 2014; Figueroa et al. 2016). The key enzyme that synthesises Tre6P and maintains the correlation between sucrose and Tre6P in Arabidopsis plants is TPS1 (Fichtner et al. 2020). Here we showed that in Arabidopsis roots, *AtTPS1* is specifically expressed in the phloem (Figure 1a,c). *AtTPPs* have a much broader expression pattern in the shoot but are predominantly expressed in the vasculature in roots (Morales‐Herrera et al. 2023; Fichtner 2025). These findings suggested an important role for Tre6P within the root vasculature.

**Figure 7:**
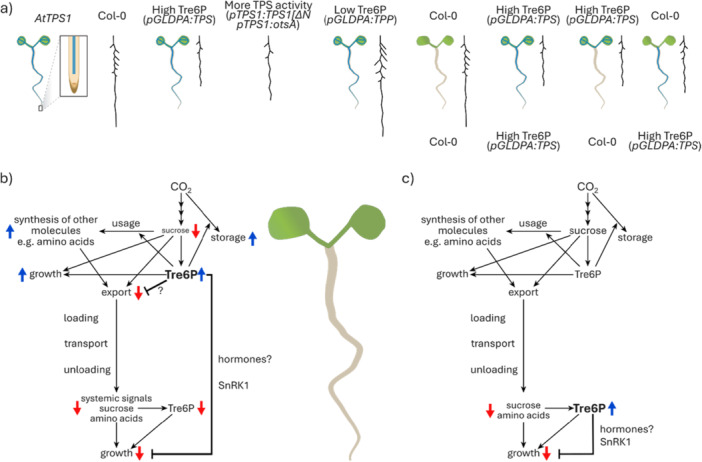
Schematic model for the role of Tre6P on root growth. (a) Summary of root growth phenotypes of lines with altered Tre6P metabolism in the vasculature. Indicated are the transgene expression patterns (blue) and the averaged root phenotypes of lines with altered vascular Tre6P levels or heterografts overexpressing *TPS* either in the shoot or root. (b) Proposed mode of action by which Tre6P systemically influences root growth. Vascular overexpression of a heterologous *TPS*, or expression of more active *TPS* variants leads to increased Tre6P synthesis. This in turn results in decreased sucrose levels, likely due to reallocation of carbon to other metabolites and enhanced shoot sink growth. The resulting decrease in sucrose levels reduces source strength and consequently lowers phloem flux to the roots, limiting the allocation of metabolites to root tissues. It remains unclear whether this effect is solely driven by reduced sucrose availability or whether Tre6P also actively regulates transport processes, for example through modulation of phloem loading and unloading. In roots, reduced supply of assimilates and other phloem transported molecules inhibits growth, while lower sucrose levels may additionally constrain Tre6P synthesis. (c) Vascular overexpression of a heterologous *TPS* in the roots may also locally inhibit root growth independently of systemic metabolic changes. This effect is likely mediated through interactions with hormonal pathways and sugar signalling components such as SnRK1 (SNF1‐related kinase 1). [Color figure can be viewed at wileyonlinelibrary.com]

Our biometric analysis of root phenotypes across multiple Tre6P mutants and grafts revealed an inverse relationship between Tre6P metabolism in the shoot vasculature and root growth (Figure 7). Interestingly, this is not always reflected by changes in steady‐state Tre6P levels. None of the lines expressing more active TPS variants or the bacterial TPS, OtsA, under the control of the native *TPS1* promoter showed detectable changes in steady‐state Tre6P levels (Supporting Information Figure S11). A similar effect was observed for the *pGLDPA:CeTPP* line, despite confirmed transgene expression (Supporting Information Figure S10a). The fact that all of these lines displayed growth phenotypes and downstream metabolic responses confirms that Tre6P dynamics are altered, even when measured Tre6P levels appear unchanged. This indicates that plants maintain Tre6P within a stable range, counteracting minor perturbations. Supporting this, sucrose levels changed as expected: lines expressing more active TPS variants had reduced sucrose, consistent with elevated Tre6P, whereas the *pGLDPA:CeTPP* line exhibited higher sucrose, consistent with decreased Tre6P. These altered sucrose levels likely act via feedback to stabilise Tre6P, in line with the Tre6P‐sucrose nexus (Yadav et al. 2014; Fichtner and Lunn 2021). This may also explain why root Tre6P levels in *pGLDPA:otsA* lines change in directions opposite to what might be expected: reduced sucrose levels in the roots inhibit Tre6P accumulation. This is further supported by qRT‐PCR data showing lower transcript levels of *TPS1* and higher transcript levels of *TPPI* in *pGLDPA:otsA* roots (Supporting Information Figure S10c). Additionally, our measurements are based on whole shoot and root tissues, and can therefore not resolve potential tissue‐specific differences (e.g., within the vasculature, as previously reported in Fichtner et al. 2021).

Previous studies have already suggested a role for Tre6P in root growth and development with a range of outcomes and observed phenotypes. For example, PR and lateral root growth were inhibited after a shift from normal to low light in Arabidopsis (Belda‐Palazón et al. 2020). Muralidhara and colleagues, however, showed that moderate perturbations in photosynthetic activity led to increased LR initiation and growth despite decreased sugar and Tre6P levels in the roots (Muralidhara et al. 2021). In contrast, Morales‐Herrera et al. (2023) found that Tre6P promotes lateral root growth in Arabidopsis grown in continuous light. In that later study, both constitutive and lateral‐root‐primordia‐specific knockouts of *AtTPPB* resulted in increased lateral root density, whereas constitutive *AtTPPB* overexpression suppressed it (Morales‐Herrera et al. 2023). In contrast, *Attppi* mutants were found to have shorter primary roots and fewer lateral roots under low nitrogen (Lin et al. 2020, 2022). These data illustrate that Tre6P can have promoting and inhibiting effects on root growth, likely dependent on the location of the Tre6P pools (LR primordia, vasculature, or other tissues), the concentration range, the nutrient status, and the signalling components in specific tissues. Our results are consistent with the observed *tppi* phenotype and short‐term perturbations in photosynthetic activity, suggesting that the function of Tre6P in the vasculature is different to the function of Tre6P within LR primordia.

### 4.2 Root Growth Modulation by Tre6P Might be a Direct Consequence of Resource Availability

By analysing the metabolic profile mediated by changes of Tre6P in the vasculature, we observed that carbon allocation was greatly affected, with large changes in sucrose, glycolytic metabolites, and organic acids (Figures 4, 5, Supporting Information Figures S9, S12, S18–S20). Besides carbon metabolism, altering Tre6P in the vasculature also had an effect on allocation of nitrogen containing metabolites like proteogenic amino acids, including major ones that are transported in the phloem (Besnard et al. 2016), like aspartate, glutamate, glutamine, serine, alanine, asparagine, and threonine (Figure 5, Supporting Information Figures S9 S10, S18–S20). The poor root growth of both ungrafted *pGLDPA:otsA* and *pGLDPA:otsA* scion wild‐type rootstock plants was associated with decreased levels of sucrose, glycolytic intermediates, and many organic acids, as well as decreased levels of many proteogenic amino acids in the roots. The large effect that Tre6P has on primary metabolism explains why the inhibition of root growth by high Tre6P could not be rescued by increasing sugar and/or nitrogen availability alone (Figures S13, S14).

The effect of Tre6P on altering nitrogen homoeostasis might be a direct effect due to Tre6P changing transcription of amino acid transporters, or indirect by altering phloem flux and dynamics, its influence on sucrose levels themselves, or activation of amino acid synthesis. Indeed, with sucrose levels driving phloem flow (Braun et al. 2014; Ludewig and Flügge 2013), and Tre6P influencing sucrose levels, Tre6P might indirectly control the allocation of other metabolites via altering sucrose levels and thereby mass phloem flow (Figure 7). If phloem transport from the shoot to the root was indeed impaired in these lines, this would limit the delivery not only of carbon and organic nitrogen but also of other essential metabolites and signals to the roots. This is supported by the fact that providing more carbon and/or organic nitrogen directly via root feeding did not rescue the root phenotype of the *pGLDPA:otsA* plants (Supporting Information Figure S14). A recent study by Xia et al. supports a role for Tre6P in macronutrient signalling. By using TRAP (translating ribosome affinity purification)‐sequencing to analyse gene expression in Arabidopsis companion cells under phosphate deficiency, they found *TPPJ* upregulated and *TPS5* downregulated, indicating that Tre6P levels and signalling respond to phosphate availability (Xia et al. 2025). Consistent with our results, these findings suggest that reduced Tre6P promotes root growth and that Tre6P coordinates growth with the allocation of carbon and macronutrients.

In addition to phloem flux and macronutrient uptake and signalling, Tre6P might also alter root growth directly, or via other metabolites and systemic signals, including hormones, SnRK1 (SNF1‐related kinase 1), or miRNAs, which may act downstream of Tre6P to regulate root growth (Figure 7).

### 4.3 Tre6P's Effects on Root Growth Might Also be a Reflection of Active Growth Regulation Through Crosstalk of Tre6P With Hormone Signalling

Strigolactone levels are responsive to changes in nutrient availability and have been implicated in the regulation of lateral root development (Yoneyama et al. 2012, 2015; Sun et al. 2014; Kapulnik et al. 2011; Koltai 2011). Interestingly, we have recently shown that there is a cross talk between Tre6P and strigolactone signalling (Fichtner et al. 2024, 2025) and that strigolactone signalling is directly antagonised by sugar availability (Barbier et al. 2015; Bertheloot et al. 2020; Patil et al. 2022). Tre6P might therefore regulate root growth not only via changes in metabolite allocation but also by directly or indirectly modifying strigolactone signalling. In fact, the synthetic strigolactone GR24 was shown to promote PR growth by increasing the number of cortical cells in the root apical meristem, while various *max* mutants deficient in strigolactone biosynthesis or signalling had the opposite phenotype (Ruyter‐Spira et al. 2011). This effect of strigolactone on root growth is thought to be mediated, amongst others, by affecting auxin transport in the root meristem (Ruyter‐Spira et al. 2013; Koltai 2015). Apart from strigolactone, auxin transport is also regulated by other factors. For example, under low energy conditions, like the ones we observed in roots of the *pGLDPA:otsA* line, bZIP11 was shown to promote the expression of *Aux/IAA3* (*Auxin/INDOLE‐3‐ACETIC ACID*), which in turn negatively regulates PIN auxin efflux carriers in the root apical meristem, inhibiting PR root growth (Baena‐González et al. 2007; Weiste et al. 2017). *bZIP11* is a potential downstream target of SnRK1, and thereby of Tre6P (Morales‐Herrera et al. 2024), linking the regulation of root growth to both carbon and hormone interactions (Delatte et al. 2011; MA et al. 2011; Kreisz et al. 2024).

Potentially other hormonal signalling pathways that are influenced by sugar availability and alterations in primary metabolism (Fàbregas and Fernie 2021), might contribute to the root growth modulation in the Tre6P accumulation lines. Indeed, transient elevation of Tre6P led to widespread changes in expression of genes involved in hormone signalling, including transcription factors involved in auxin, gibberellic acid, ethylene, abscisic acid, and jasmonic acid signalling pathways (Avidan et al. 2024).

## 5 Conclusions

In summary, our data shows that vascular Tre6P levels modulate shoot and root growth by balancing the C/N ratio by altering the allocation and utilisation of resources. Tre6P signalling thereby shows cell and tissue specific effects. Modifying Tre6P levels in a tissue and cell‐specific manner specifically within roots and shoots could therefore be a promising strategy for future crop breeding approaches aiming at optimising shoot and root growth parameters.

## Supporting information


**Figure S1:** Expression pattern of TPS1 in Arabidopsis roots.
**Figure S2:** Expression pattern of *TPS1* in Arabidopsis roots.
**Figure S3:** Expression pattern mediated by the *GLYCINE‐DECARBOXYLASE P‐SUBUNIT A (GLDPA)* promoter from *Flaveria trinervia* in Arabidopsis.
**Figure S4:** Changing vascular Tre6P levels alters the root system architecture.
**Figure S5:** Tre6P consistently alters root development.
**Figure S6:** Visual representation of the average root system architecture (RSA) in Arabidopsis plants with different vascular Tre6P levels.
**Figure S7:** Confirmation of the genotypic background of the *CRISPR TPS1[ΔN5‐90]* line.
**Figure S8:** Changing vascular Tre6P levels alters the rosette diameter.
**Figure S9:** Metabolite levels over the course of a day in Arabidopsis lines with altered Tre6P levels in the vasculature.
**Figure S10:** Vascular overexpression of *TPS* or *TPP* alters the natural expression of *Tre6P* pathway genes.
**Figure S11:** Metabolite levels in Arabidopsis lines overexpressing modified variants of *TPS1* or a bacterial *TPS*.
**Figure S12:** Individual amino acid levels over the course of a day in Arabidopsis lines with altered Tre6P levels in the vasculature.
**Figure S13:** Increasing carbon availability by altering the photoperiod cannot rescue the root growth defect of lines with elevated Tre6P levels in the vasculature.
**Figure S14:** Supplying carbon and/or organic nitrogen cannot rescue the root growth defect of lines with elevated Tre6P levels in the vasculature.
**Figure S15:** Changing Tre6P levels alters metabolite allocation.
**Figure S16:** Root morphology of grafted Arabidopsis seedlings with root or shoot vasculature specific expression of a bacterial TPS (otsA).
**Figure S17:** The shoot growth in seedlings with alterations in Tre6P in the vasculature of shoots, or roots is consistent with their root growth phenotype.
**Figure S18:** Systemic changes in vascular Tre6P levels are accompanied by widespread changes in primary metabolism.
**Figure S19:** Tre6P systemically alters allocation of metabolites between shoots and roots.
**Figure S20:** Systemic changes in vascular Tre6P levels are accompanied by widespread changes in primary metabolism.
**Table S1:** single guide RNA (sgRNA) sequences used for the generation of the *CRISPR TPS1[ΔN_5‐90_]* line.
**Table S2:** Oligonucleotide primers used for genotyping the deletions of the N‐terminal domain in the *TPS1[ΔN]* lines.
**Table S3:** Oligonucleotide primers used for gene expression analyses of *TPS* and *TPP* genes in the *pGLDPA:otsA.5* and the *pGLDPA:CeTPP.4* lines.

Supporting File 1

Supporting File 2

Supporting File 3

## Data Availability

The data that support the findings of this study are openly available at https://git.nfdi4plants.org/hhu‐plant‐biochemistry/goebel_et_al_2026. https://doi.org/10.60534/jkrz2-e1n55. We thank DataPLANT for support. [Correction added on 3 July 2026, after first online publication: The Data Availability Statement has been updated.]
